# Giant Cell Arteritis Masquerading as Tooth Pain: A Case Report

**DOI:** 10.7759/cureus.57836

**Published:** 2024-04-08

**Authors:** Srishti Manocha, Pranav Kataria, Nilansh Kataria, Rajesh Manocha

**Affiliations:** 1 General Dentistry, Private Practice, Ottawa, CAN; 2 Department of Periodontics, University of Connecticut Health, Farmington, USA; 3 Department of Internal Medicine, MedStar Washington Hospital Center, Washington, DC, USA; 4 Department of General Medicine, ESIC Medical College and Hospital, Faridabad, IND

**Keywords:** dental emergency, difficult diagnosis, headache, jaw pain, role of dentist, giant cell arteritis (gca)

## Abstract

Giant cell arteritis (GCA) is a form of vasculitis characterized by symptoms that often lead a patient to consult a general dentist. Its rarity in the dental setting and serious life-altering effects make it a formidable diagnosis. We discuss a case of a 60-year-old female with GCA presenting with primary symptoms of excruciating tooth and jaw pain on the left side. We also report secondary symptoms of headache and partial vision loss and engage in a review of the relevant literature. Jaw pain, unexplained toothache, or tissue necrosis in patients aged over 50 years can be misdiagnosed as joint arthritis or temporomandibular disease (TMD), which could lead to severe consequences. Accurately diagnosing this ophthalmic emergency is critical for implementing therapy promptly and preventing ischemic complications. Dentists should maintain a high index of suspicion about its signs and symptoms, which will aid in making an early diagnosis and prompt referral.

## Introduction

Giant cell arteritis (GCA), also known as temporal arteritis or Horton’s arteritis [1], is a granulomatous vasculitis typically affecting the large- and medium-sized arteries, including the aorta, branches of the ophthalmic artery, and extracranial branches of the carotid arteries [2]. As a critical diagnosis, a high index clinical suspicion is required to diagnose the condition early before it progresses to a stage of permanent vision loss [3]. Its pathogenesis is T-cell driven. Its name refers to the giant cells seen histologically in the affected vessels [4,5]. It is the most common form of vasculitis seen in North America, and its classical presentation is observed in individuals over the age of 50 years. Presenting symptoms may include headaches (76%), tongue and jaw pain, stroke, and vision loss due to the inflammation of vessels [6]. Hazelman and Jones have described three GCA cases, where the diagnosis was delayed by the dentist, which led to a delay in the administration of corticosteroid therapy, resulting in life-threatening consequences [7].

This case report aims to raise awareness among dental professionals about the presentation-related characteristics, signs and symptoms, and demographic patterns of GCA. We believe our findings will enable dentists to avoid misdiagnosing such cases for endodontic involvement or temporomandibular disease (TMD) due to the similarities in presentations.

## Case presentation

The patient was a 60-year-old healthy Caucasian female with no significant medical history who presented to the dental office complaining of severe pain on the left side of her face. Associated symptoms included headache in the frontotemporal region and blurry vision that had started around three weeks back. Her pain was centered in the teeth and jaw on the left side and radiated to the ipsilateral temple and neck, thus prompting her to visit our dental office. On extraoral examination, the left temporal aspect of her face was tender and erythematous, whereas the right temporal aspect seemed pale but not tender to palpation.

On intraoral examination, no obvious tooth decay was found, and the gingiva appeared pink and firm. No tissue necrosis was noted. Tenderness on percussion was negative on all teeth on the upper left and lower left quadrants. Palpation in the vestibular area was also not tender. A series of periapical radiographs of her upper and lower left quadrant teeth were done to rule out interproximal decay or periapical pathology. No pathology was detected on the X-rays. A prompt referral was made to the emergency room along with her dental x-rays and other findings.

In the ED, vitals were significant for elevated blood pressure, which, combined with other signs and symptoms such as headache and blurry vision led the clinicians to consider a differential diagnosis of arteritis. A blood test was ordered along with the initiation of oral prednisone 50 mg daily due to high suspicion of vasculitis or polymyalgia rheumatica.

**Figure 1:**
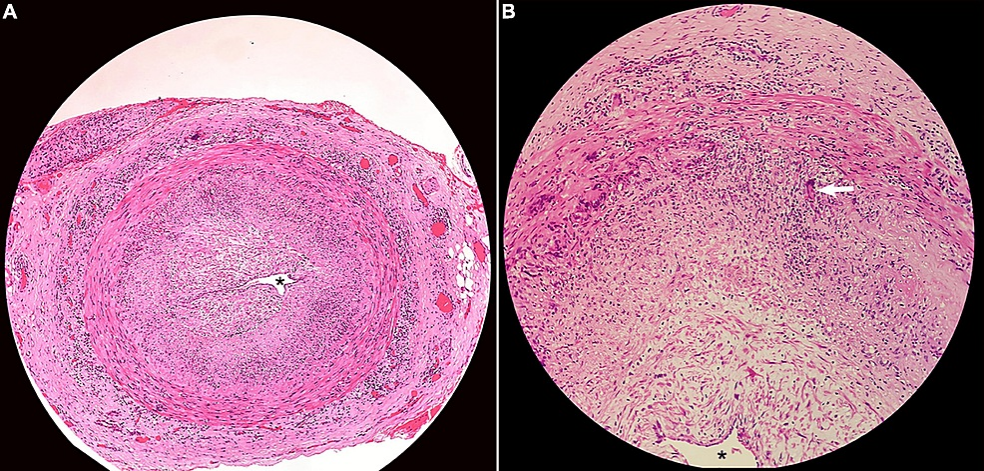
Temporal artery biopsy A. Hematoxylin and eosin stain (objective: 4x) - narrowing of the temporal artery, where the asterisk (*) demonstrates the narrow lumen allowing for minimal blood flow. B. Hematoxylin and eosin stain (objective: 10x) - the arrow shows the presence of giant cells along with lymphocytes and histiocytes in the cross-section of the temporal artery

Significant lab investigations included elevated erythrocyte sedimentation rate (ESR) at 58mm/hr and elevated C-reactive protein (CRP) at 54.6mg/mL, demonstrating systemic inflammation. The rheumatologist ordered a temporal artery biopsy (TAB), in which the left temporal artery demonstrated focal areas of inflammation, narrowing of the lumen of the blood vessel, and the presence of giant cells, findings consistent with the diagnosis of arteritis (Figures 1A, 1B). TAB is considered the gold standard for the diagnosis of GCA. It should be noted that the diagnosis was made more than 30 days after the onset of the first symptoms in the patient; however, glucocorticoid therapy was initiated promptly and not withheld while awaiting the results of the biopsy [8].

The patient was kept under review by her rheumatologist and neurologist. CRP levels decreased substantially to 9.3 mg/L, following the administration of glucocorticoids, and headaches dissipated completely. There was no evidence of stroke on the contrast CT. She, however, experienced the side effects of prolonged use of steroids, including immunosuppression, and hence was vaccinated for pneumonia, shingles, and flu and tested for tuberculosis (TB). The patient has gained 20 lbs in four months since the incidence of GCA, tapered down her prednisone dose to 20 mg daily, and is now monitored through routine hematological assessments and CT scans.

This case, although rare, illustrates the importance of dentists considering GCA as a potential diagnosis when encountering cases mimicking unexplained dental pain, which can save patients from life-threatening consequences.

## Discussion

GCA as we know it is a systemic vasculitis first described in the English language in 1890, when an individual suffering from the condition and could not wear their hat due to severe pain on the side of the head from inflamed temporal arteries was examined [9]. It was further analyzed histopathologically in 1932 by Horton, hence bearing the eponyms of Horton’s arteritis and temporal arteritis [10]. The overall incidence of this condition ranges from 2-30 per 100,000 individuals with an increased propensity of occurrence in individuals over the age of 50 years. The Caucasian race of Scandinavian and European descent seems to be a significant predisposing factor. The condition has a lower prevalence among the African American and Asian populations [11,12].

The immunological events that lead to the clinical manifestations of GCA are unclear and have been hypothesized to occur in a staged manner. The first phase involves a nonspecific acute response to stress and injury. It is not antigen-driven and is characterized by vague markers of inflammation such as fevers, myalgia, and anorexia. This is followed by the second response, which is aggressively antigen-driven and focused on the arterial walls, causing occlusion and ischemic symptoms of GCA [13]. Reports showing a link between dental symptoms and a properly managed diagnosis of GCA are scarce in the literature. This study is unique in that the severity of our patient’s tooth and jaw pain on the left side masked other symptoms of headache and blurry vision, prompting her to see a dentist.

According to the American College of Rheumatology, the presence of three out of the five criteria, as described in Table 1, in a patient enables the diagnosis of GCA with a sensitivity of 93% and specificity of 91%. In our patient, an elevated ESR, age of onset, and abnormal TAB findings confirmed the diagnosis.

**Table 1:** The American College of Rheumatology criteria for the diagnosis of GCA* *[14] GCA: giant cell arteritis

Serial no.	Criteria
1	Age more than 50 years
2	New-onset headache
3	Temporal artery abnormality
4	Erythrocyte sedimentation rate (ESR) >50 mm/hr
5	Histologic evidence of arteritis on temporal artery biopsy (TAB)

According to one study, 25%-50% of patients with untreated GCA experienced contralateral loss of vision over the following one to two weeks [15]. This highlights the importance of early recognition and treatment of GCA to prevent permanent vision loss. Another common dental presentation in GCA patients is jaw pain, noted in 31% of cases in the studies reviewed [16]. It is important to differentiate between jaw claudication that happens in GCA versus TMJ jaw pain, as described in Table 2.

**Table 2:** Differences between jaw pain associated with TMJ and GCA GCA: giant cell arteritis; TMJ: temporomandibular joint

TMJ jaw pain	Jaw claudication - GCA
1. Occurs between 25-44 years	1. Occurs after 50 years
2. Pain happens while chewing	2. Pain occurs a few minutes after chewing
3. Associated with trismus, muscle spasm	3. Associated with ipsilateral swelling, potential soft tissue necrosis
4. Usually localized to the jaw, ear, and temple	4. May be associated with symptoms of headache, fever, and anorexia

## Conclusions

GCA is an important diagnosis in dentistry due to its infrequent presentation, rapid progression, and life-altering nature. Its signs and symptoms can occasionally present as unexplained dental pain as seen in this case. General dental practitioners should maintain a high level of suspicion regarding this condition when encountering patients over 50 years of age, as it is paramount to make an early diagnosis and speedy referral, preventing dire ischemic consequences in these patients.
